# Enzymatic synthesis of long double-stranded DNA labeled with haloderivatives of nucleobases in a precisely pre-determined sequence

**DOI:** 10.1186/1471-2091-12-47

**Published:** 2011-08-24

**Authors:** Ireneusz Sobolewski, Katarzyna Polska, Agnieszka Żylicz-Stachula, Joanna Jeżewska-Frąckowiak, Janusz Rak, Piotr Skowron

**Affiliations:** 1 Department of Chemistry University of Gdańsk, Sobieskiego 18, 80-952 Gdańsk, Poland

## Abstract

**Background:**

Restriction endonucleases are widely applied in recombinant DNA technology. Among them, enzymes of class IIS, which cleave DNA beyond recognition sites, are especially useful. We use BsaI enzyme for the pinpoint introduction of halogen nucleobases into DNA. This has been done for the purpose of anticancer radio- and phototherapy that is our long-term objective.

**Results:**

An enzymatic method for synthesizing long double-stranded DNA labeled with the halogen derivatives of nucleobases (Hal-NBs) with 1-bp accuracy has been put forward and successfully tested on three different DNA fragments containing the 5-bromouracil (5-BrU) residue. The protocol assumes enzymatic cleavage of two Polymerase-Chain-Reaction (PCR) fragments containing two recognition sequences for the same or different class IIS restriction endonucleases, where each PCR fragment has a partially complementary cleavage site. These sites are introduced using synthetic DNA primers or are naturally present in the sequence used. The cleavage sites are not compatible, and therefore not susceptible to ligation until they are partially filled with a Hal-NB or original nucleobase, resulting in complementary cohesive end formation. Ligation of these fragments ultimately leads to the required Hal-NB-labeled DNA duplex. With this approach, a synthetic, extremely long DNA fragment can be obtained by means of a multiple assembly reaction (n × maximum PCR product length: n × app. 50 kb).

**Conclusions:**

The long, precisely labeled DNA duplexes obtained behave in very much the same manner as natural DNA and are beyond the range of chemical synthesis. Moreover, the conditions of synthesis closely resemble the natural ones, and all the artifacts accompanying the chemical synthesis of DNA are thus eliminated. The approach proposed seems to be completely general and could be used to label DNA at multiple pre-determined sites and with halogen derivatives of any nucleobase. Access to DNAs labeled with Hal-NBs at specific position is an indispensable condition for the understanding and optimization of DNA photo- and radio-degradation, which are prerequisites for clinical trials of Hal-NBs in anticancer therapy.

## Background

Class IIS restriction endonucleases (REases) are widely applied in recombinant DNA technology. In a series of pioneer studies, Szybalski et al. [1] showed that class-IIS REases offer novel possibilities for DNA manipulation, for example, a "universal restriction endonuclease", cleaving a pre-determined sequence in single-stranded DNA with the assistance, *inter alia*, of hairpin oligodeoxyribonucleotides (oligo) [2,3], Achilles' Heel Cleavage of DNA [4,5], a DNA fragment amplification vector [6], and chimeric repressor-REase enzymes [7]. Here we use class IIS enzymes to precisely incorporate modified nucleobases into DNA in order to enhance its photo- and radiosensitivity.

Substituting a hydrogen atom of a nucleic base (NB) with a halogen atom leads to a minor change in the NB structure. As a result, DNA containing halogen-substituted nucleobases (Hal-NBs) possesses the same structural features as the native biopolymer. Owing to the photo- and radiosensitivity of Hal-NB, however, such modified DNA becomes more susceptible to damage by ionizing radiation and more sensitive to UV light. Therefore, halogen-substituted uracils (5-XU) have been used in the past as structural probes of the nucleic acid structure [8-10] and for mapping protein-nucleic acid interactions [11-14]. 5-bromouracil (5-BrU) has also been employed in studies of long-distance electron transfer in DNA [15]. The attachment of an electron to 5-BrU triggers the quantitative release of bromide anions and uracil, a process that is easily evaluated. Since the number of 5-BrU/electron reactions can be monitored, the maximum average electron migration distance along the helix can be estimated [16]. Recently, Cecchini et al. [17] demonstrated that the irradiation of double-stranded DNA containing 5-BrU with ionizing radiation leads to interstrand cross-links, one of the most toxic types of DNA damage. The toxicity of DNA cross-links is probably the result of two mechanisms: one prevents strand separation, thus inhibiting both transcription and replication, the other can induce mutations and DNA rearrangements as a result of the repair process [17]. It has also been shown that irradiation of 5-bromocytosine (5-BrC) [18-20] labeled DNA with near-UV light brings about intrastrand photo-cross-linking: adducts are formed photochemically between 5-BrC and adjacent nucleobases.

Incorporation of 5-BrU in DNA increases the biopolymer's susceptibility to single- and double-strand breakage induced by ionizing radiation [21] and influences the rate of DNA repair of potentially lethal damage [22]. Among the main products of water radiolysis are low-energy electrons that can attach to bromouracil [17]. The unstable bromouracil anion is stabilized when the bromine anion is detached, which causes the very reactive uracil-5-yl radical to form. This species can attack the C2' and C1' centers of the adjacent 2-deoxyribose, thereby inducing DNA strand cleavage. It is also assumed that the same uridyl radical forms as a result of interaction between UV-radiation and DNA labeled with 5-bromouracil [23,27]. The preliminary steps of the photochemical process are probably related to photo-induced electron transfer between electronically excited 5-bromouracil and the adjacent nucleobase [25,26]. This conclusion was suggested by the experimentally observed strong dependence of the extent of damage on the base sequence [27-29].

Since the structural changes associated with the incorporation of a halogen atom into a nucleic acid base are only minor, nucleotides containing Hal-NBs can be used by a cell for DNA biosynthesis (the cellular enzymatic system does not radically differentiate between Hal-NBs and natural nucleotides) [30]. Furthermore, the incorporation of Hal-NBs in DNA sensitizes the biopolymer to UV and ionizing radiation. It seems, therefore, that Hal-NBs could be employed in the therapy of cancer diseases. It has already been reported that 5-BrU increases the likelihood of cells being damaged by ionizing radiation [31]. The enhanced response of non-hypoxic tumor cells to radiation in the presence of bromodeoxyuridine, iododeoxyuridine and fluorodeoxyuridine has been recognized in several clinical trials [32-34]. Some clinical studies have also reported the radiosensitization of malignant brain tumors by bromodeoxyuridine [35].

With such properties, Hal-NBs are potentially potent anticancer drugs in radio- and phototherapy. However, without an understanding of the molecular mechanisms leading to DNA breakage their rational use is not possible. Hence, before these substances can be implemented in clinical practice, comprehensive, multidisciplinary studies need to be carried out in order to elucidate the consecutive stages of the process leading to DNA damage induced by UV light or an excess electron.

If the photo- and radiochemical studies aiming at the understanding and optimization of DNA degradation process are to yield reliable results, we need access to DNA fragments of pre-defined sequences labeled with Hal-NBs at precisely determined positions. Furthermore, relatively short double-stranded DNA (from tens to hundreds of base pairs) is known to behave more like a "rigid rod" [36]. On the other hand, DNA molecules *in vivo *achieve lengths of from several thousand to hundreds of millions of base pairs. Hence, short synthetic DNA fragments seem to be quite a crude model of natural DNA, since the effects due to folding (e.g. supercoiled DNA) could profoundly affect the susceptibility to damage of the biopolymer's chemical bonds. So far, all studies of damage to Hal-NB-labeled DNA have focused on short oligo fragments - 30-40 base-pairs (bp) at the most. In this work we describe a new technique of DNA labeling for synthesizing DNA fragments of up to several hundred base pairs. This target has been achieved by employing the site-directed introduction of nucleotide derivatives into dsDNA molecules with the use of a multi-enzymatic complex. Such long DNA duplexes behave in a manner similar to that of natural DNA and are beyond the range of efficient chemical synthesis. Moreover, bacterial thermostable polymerases are employed in DNA synthesis; consequently, the conditions of DNA synthesis closely resemble the natural ones, and artifacts accompanying chemical synthesis (impurities resulting from side-products, uncontrolled modifications, heterogeneity of DNA length, etc.) are thus eliminated. Although the nature of these trace contaminants has not been evaluated, gene cloning scientists are well aware that synthetic DNA tends to cause far greater problems in enzymatic manipulation than DNA obtained from a native source or in a PCR reaction. This also applies to ligation efficiency.

In the following we present details of the method specially designed to prepare long DNA duplexes labeled with Hal-NBs.

## Results and discussion

### General idea

**Figure 1:**
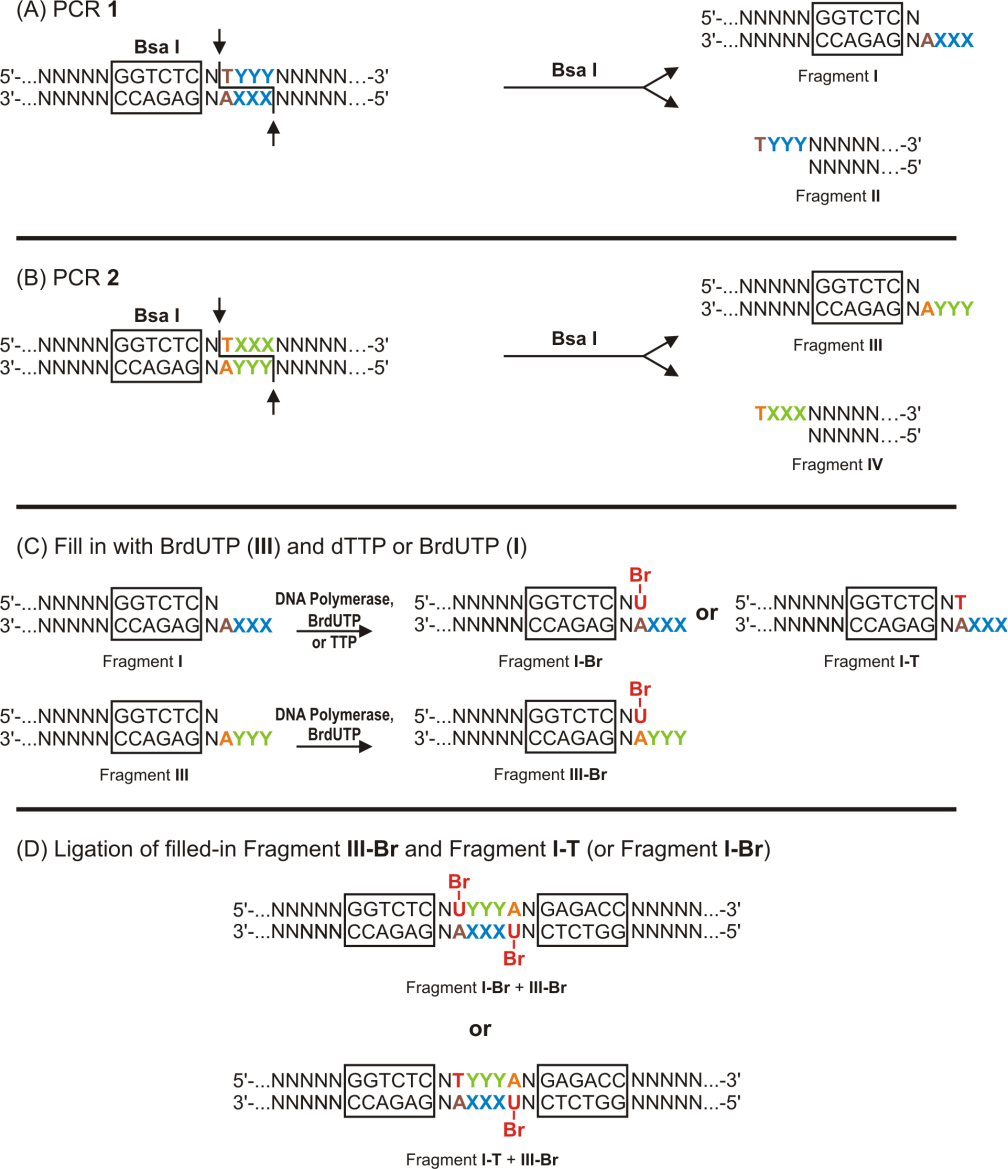
**Diagram of single- and double-label incorporation with pinpoint precision internally within long DNA molecules**. Br-U symbolizes incorporated 5-bromodeoxiuridine; N symbolizes unspecified nucleotides. * complementarity between the three bases within the overhang (XXX/YYY) is necessary

The incorporation of one or two molecules of 5-BrdU per molecule of synthetic duplex taking place in the above-mentioned process is illustrated schematically in Figure 1. Let us assume that two double-stranded DNA fragments (**I-T **and **III-Br**, Figure 1C) possessing 3-nt complementary sticky ends are available. One of these fragments (**III-Br**) contains 5-BrdU instead of thymidine in the base pair preceding the cohesive end. After the ligation of **I-Br **with **III-T**, the final product is synthesized, i.e. double-stranded DNA internally labeled with 5-BrdU. **I-T **and **III-Br **can be obtained from fragments **I **and **III **(Figure 1C) using a DNA polymerase and dTTP or BrdUTP, respectively. The first base of the initially non-complementary 4-nt cohesive ends of fragments **I **and **III **has to be adenine if a DNA fragment labeled with 5-BrdU is to be synthesized. The filling of the non-complementary strand with thymidine or 5-bromouridine (single- or double labeling of both strands) produces two complementary, 3-nt sticky ends. If, for instance, the 5'-YYY sequence (the lower strand of the final product) appears at the cohesive end of **III-Br**, the 5'-XXX sequence (the upper strand of the final product) has to be generated in **I-T**, where X and Y are the complementary bases. Thus, the 4-nt cohesive ends generated by a class-IIS REase cannot be cross-ligated between duplexes **I **and **III **unless the first base of the 4-nt cohesive end is filled with dTTP (or BrdUTP) (Figure 1D).

So, in order to obtain a DNA fragment with 5-BrdU incorporated into it, two double stranded duplexes (PCR1 and PCR2, Figure 1AB) have to be synthesized with appropriately located terminal sites sensitive to class-IIS REases. These duplexes are then used to generate sub-duplexes by BsaI digestion, yielding four fragments: I, II, III, and IV. Filling duplexes **I **and **III **with BrdUTP (resulting in **I-Br **and **III-Br**, Figure 1C) yields an internally double labeled DNA molecule containing two BrdU residues separated by 3 bp.

**Figure 2:**
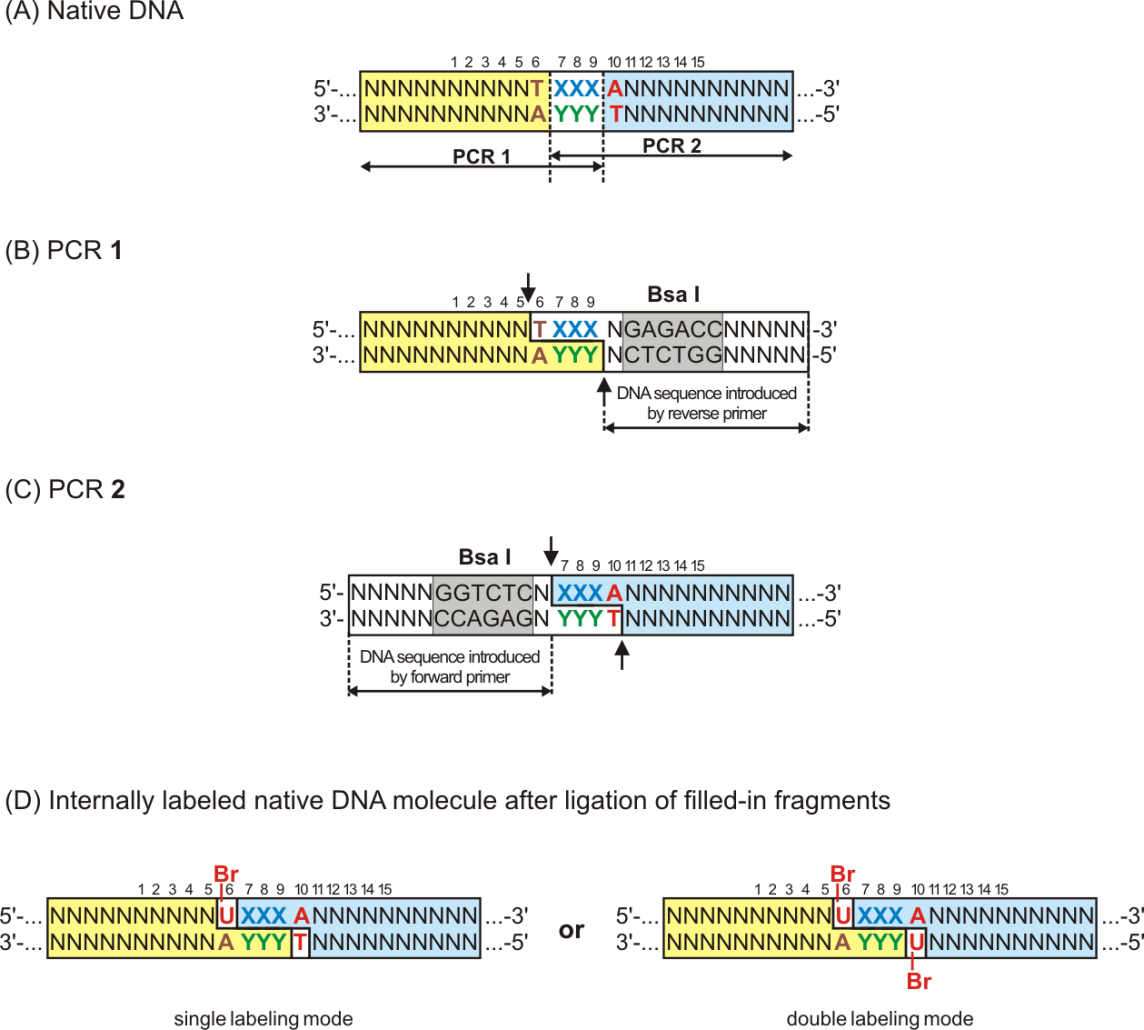
**Diagram of internally labeled native-sequence hybrid DNA molecule generation**. To obtain a labeled native DNA sequence the following sequence requirements must be fulfilled: * in the seventh (X) and/or ninth position (Y) a non-A nucleotide is required depending on the labeling mode. * complementarity between the three bases within the overhang (positions 7-9; XXX/YYY) is necessary. * A/T in the tenth position is not required for a single DNA labeling. Br-U symbolizes incorporated 5-bromodeoxiuridine; N symbolizes unspecified nucleotides

In principle, the same or different class-IIS REases, producing 5' protruding 4-nt cohesive ends, could be used for the cleavage of PCR1 and PCR2. After minor modification, also other class-IIS REases producing 5' protruding cohesive ends of the length 2-nt or longer can be used. The choice of class-IIS REase used will also be dictated by the preferred absence of its cognate site within the DNA segment to be amplified as well as by its desired enzymatic parameters. However, the presence of the same class-IIS site within the DNA molecule does not preclude application of the method. Statistically only 1 in 256 4-nt cohesive ends in the cleavage site will be identical. Because of the uniqueness of cohesive ends generated by class-IIS REases, only intended hybrid molecules should be assembled. However, if the cohesive ends have consecutive adenines (Figure 2; in the seventh (X) and ninth (Y) position) resulting ends will not be able to ligate after filing them with BrdUTP. In case of a singly labeled DNA, on average 1 in 4 of 4-bp cleavage sites sequences will block the assembly reaction. Hence, for the 6-bp class IIS REase recognition site (on average one every 4096 bp), flanked by 4-bp cleavage site, the maximum length of the final labeled molecule is limited to the assembly of 3 permissive combinations (C, G or T in the crucial position). Thus the maximum length of the labeled DNA molecule amounts to 4096 × 3 = 12 288 bp. In order to overcome limitation imposed by the presence of the internal recognition sites one may select those REases, which recognize longer DNA sequences. For example, SapI REase recognizes 7-bp sequence: GCTCTTC(N1/4), thus cleaving on average every 16 384 bp [37]. Another solution of the problem could be the 2-step methylation method for extension of a recognition site of a class-IIS REases [38].

There are two alternative modes of assembling hybrid DNA molecules. In the first mode, native sequence hybrid DNA molecules are generated when class-IIS sites are oriented toward the 3' ends of the primers used and are adjacent to the beginning of the original, native sequence to be re-joined. 5' portions of the primers along with the class-IIS sites are cut off during the procedure, leaving only 4-nt cohesive ends, which are part of the original DNA template (Figure 2). When the natural sequences are to be labeled, they must be in the form of 5'-TXXXN-3', where the first X cannot be T and N cannot be identical to the last X (in the single position labeling variant) or the sequence must be in the form of 5'-TXXXA-3', where the first X cannot be T and the last X cannot be A (in the double position labeling variant). The bases in the 7-9 positions (marked as Xs) do not have to be identical (Figure 2A). It is important to remember during primers design, that incorporated class-IIS REase sites need to be properly positioned, e.g. offset by one base in the two primers to give 5'-XXXA (or 5'-XXXN for single labeling variant) and 5'-YYYA overhangs, to avoid incorporation of an additional base into the native sequence (Figure 2D).

The second mode is based on either the 5'- or the 3'- orientation of the class-IIS REase sites, where additional sequences are incorporated from the primers used (with or without the class-IIS sites used) (Figure 1). Should additional sequences be incorporated, the procedure will result in internally labeled mutant hybrid molecules. The potential applicability extends beyond an incorporation of Hal-Nbs for DNA damage studies, but can be used to study protein-DNA interaction by, for example, the precise incorporation of modified bases into the DNA-interacting protein recognition sites present on long DNA molecules.

### Design of hybrid, BrdU-labeled DNA molecules with the use of BsaI REase

A specific class-IIS enzyme BsaI recognizes 5'-GGTCTC-3' DNA sequence and cleaves downstream at 1 nt (top strand) and 5 nt (bottom strand) at any sequence, leaving complementary "sticky" ends. BsaI was selected for this study as it recognizes DNA relatively infrequently - on average every 4096 bp, cleaves DNA to completion and is a robust thermophilic enzyme. Processing duplexes with the enzyme generates two fragments, I and II, for the first duplex (PCR 1), and two fragments, III and IV, for the second one (PCR 2) (Figure 1 AB). Since both double-stranded duplexes possess the class-IIS REase (BsaI) site, the tetranucleotide "sticky" ends produced by BsaI cleavage can be pre-programmed to have any sequence. The sequence of the cohesive ends has been devised in such a way that the first base, immediately adjacent to the double stranded portion of the DNA fragment (thus closest to the class-IIS BsaI recognition site), is the same for both the first and the second duplex (in the case of BrdU incorporation, it is the complementary base A), whereas the following 3-nt are reverse complements: 5'-GGG and 5'-CCC. Thus, the cohesive ends generated by BsaI are 5'-GGGA and 5'-TCCC for duplex 1, and 5'-CCCA and 5'-TGGG for duplex 2. Hence, the cohesive ends generated cannot be cross-ligated between duplexes 1 and 2 unless the first base of the 4-nt cohesive end is filled with dTTP (or BrdUTP), generating complementary 5'-CCC and 5'-GGG single stranded DNA sequences (Figure 1C). Cleaved PCR fragments not filled with BrU are effectively excluded from the ligation reaction, as no compatible cohesive ends are produced. Thus, to activate the final step, the missing thymidine in fragment III needs to be complemented with BrdU and the missing thymidine (or BrdU for the purpose of double labeling) in fragment I. Ligation of fragments I and III produces the new double stranded duplex, containing one or two precisely incorporated internal BrdU residues (Figure 1D). In general, the procedure can be extended, so that in one production cycle duplexes can be prepared with several different specific sequences cleaved by different enzymes, incorporating a label at more than one pre-defined location or incorporating different labels in the same DNA molecule. We chose 5'-CCC/5'-GGG cohesive ends to increase annealing, and therefore to achieve more efficient ligation. Nevertheless, the method is universally applicable to any asymmetric cohesive ends, as long as complementarity is generated upon partial filling. The mutations accumulating in the PCR step of preparing DNA to be modified may constitute a limitation to the method if an excessive number of cycles is being used. It would therefore be beneficial to optimize every labeling reaction at the initial PCR stage as well, in order to minimize the effective number of cycles needed. The polymerase used imposes a limitation on a modified nucleotide incorporated into DNA. Every polymerase/label combination therefore needs to be verified experimentally.

### Assessing various DNA polymerases and assembly reaction conditions for the ability to incorporate BrdU

**Figure 3:**
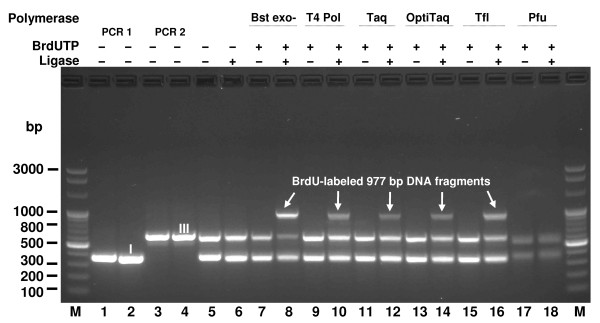
**Assessment of various DNA polymerases for their ability to incorporate BrdU**. Complete and incomplete specific incorporation reactions (Figure 1) were carried out with 5 DNA Polymerases: Bst exo^- ^(thermophilic), T4 (mesophilic), Taq (thermophilic), OptiTaq (thermophilic blend) and Pfu (hyperthermophilic) in the presence of BrdUTP. Lanes M, Perfect 100 bp Ladder; lane 1, PCR 1 fragment (379 bp); lane 2, BsaI-cleaved PCR 1 fragment; lane 3, PCR 2 fragment (625 bp); lane 4, BsaI-cleaved PCR 2 fragment; lane 5, BsaI restriction fragments: I (363 bp) and III (609 bp). Lanes 6-18 reactions with specified DNA Polymerases: lane 6, restriction fragments: I and III, T4; lane 7, restriction fragments: I and III, Bst exo^- ^; lane 8, restriction fragments: I and III, Bst exo^- ^, T4 DNA Ligase; lane 9, restriction fragments: I and III, T4; lane 10, restriction fragments: I and III, T4, T4 DNA Ligase; lane 11, restriction fragments: I and III, Taq; lane 12, restriction fragments: I and III, Taq, T4 DNA Ligase; lane 13, restriction fragments: I and III, OptiTaq; lane 14, restriction fragments: I and III, OptiTaq, T4 DNA Ligase; lane 15, restriction fragments: I and III, Tfl; lane 16, restriction fragments: I and III, Tfl, T4 DNA Ligase; lane 17, restriction fragments: I and III, Pfu; lane 18, restriction fragments: I and III, Pfu, T4 DNA Ligase. ^I, III ^BsaI restriction fragments numbered as in Figure 1.

The method described here was used as a model for further anti-cancer therapy studies; it was not limited to the use of BrdUTP as a cytotoxic compound when incorporated into DNA. It is expected that the different labels present in modified dNTPs will be utilized with various affinities by DNA Polymerases, thus resulting in incorporation into DNA with greatly varying efficiency. Reaction limitations may also arise from the substrate requirements of DNA Ligase. Figure 3 shows labeling reactions performed under demanding conditions - double BrdU residues incorporated in close proximity using various DNA polymerases. The conditions of double labeling were chosen to determine whether DNA Ligase is capable of joining separate DNA molecules, containing unnatural bases within strand nicks, just 3 bp apart. Clearly, DNA Ligase can utilize such unusual substrates. Moreover, we explored the possibility of increasing label density in DNA for the purpose of future studies of the enhanced sensitization of DNA and stimulation of double strand breakage following irradiation of neighboring BrdU residues in DNA: after incorporation, the modified Hal-modified residues are separated by only 3 bp.

**Figure 4:**
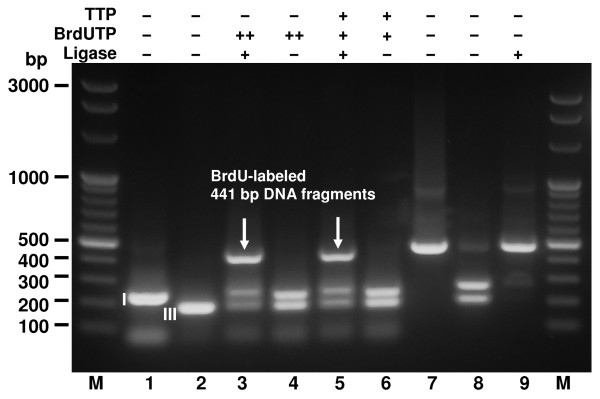
**Incorporation of double and single BrdU residues by Bst exo^- ^DNA Polymerase into the 441 bp hybrid molecule**. Incorporation reactions using BrdUTP alone or in combination with dTTP were carried out with Bst exo^- ^DNA Polymerase. Lanes M, Perfect 100 bp Ladder (selected bands marked); lane 1, 260 bp BsaI-cleaved PCR (restriction fragment I); lane 2, 208 bp BsaI-cleaved PCR (restriction fragment III); lane 3, BrdUTP-filled restriction fragments I and III, T4 DNA ligase; lane 4, BrdUTP-filled restriction fragments I and III; lane 5, dTTP-filled restriction fragment I and BrdUTP-filled restriction fragment III, T4 DNA ligase; lane 6, dTTP-filled restriction fragment I and BrdU-filled restriction fragment III. Lanes 7-9, controls of enzymes functional purity: lane 7, control PCR fragment with internal BsaI site; lane 8, BsaI-cleaved control PCR fragment; lane 9, BsaI-cleaved control PCR fragment after addition of T4 DNA Ligase; lane M, Perfect 100 bp Ladder. ^I, III ^BsaI restriction fragments numbered as in Figure 1.

**Figure 5:**
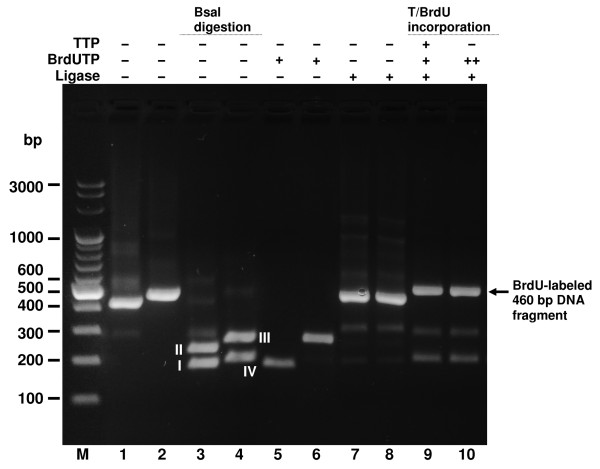
**Incorporation of double and single BrdU residues by Bst exo^- ^DNA Polymerase into the 466 bp hybrid molecule**. Incorporation reactions using BrdUTP alone or in combination with dTTP were carried out with Bst exo^- ^DNA Polymerase. Lanes M, Perfect 100 bp Ladder (selected bands marked). Enzyme purity and reaction steps controls: lane 1, uncut 437 bp PCR fragment amplified from pGCN1 plasmid; lane 2, uncut 480 bp PCR fragment amplified from pGCN2 plasmid; lane 3, BsaI-cut 437 bp fragment; lane 4, BsaI-cut 480 bp fragment; lane 5, BsaI restriction fragment I (191 bp) filled in with BrdUTP isolated from agarose gel; lane 6, BsaI restriction fragment III (270 bp) filled in with BrdUTP isolated from agarose gel; lane 7, BsaI-cut 437 bp fragment, purified and back-ligated; lane 8, BsaI-cut 437 bp fragment, purified, incubated with Bst exo- DNA Pol without dNTPs and back-ligated. Incorporation reaction: lane 9, fragment I (191 bp) filled in with dTTP, ligated to BrdU-labeled fragment III (270 bp); lane 10, fragment I (191 bp) filled in with BrdUTP, ligated to BrdU-labeled fragment III (270 bp). ^I, III ^BsaI restriction fragments numbered as in Figure 1.

The evaluation of polymerases shows clearly that the *Bacillus stearothermophilus *DNA Polymerase exo^- ^C-terminal fragment exhibits the highest final yield of the generation hybrid 977 bp DNA molecule (Figure 3, lane 8), while the mesophilic T4 bacteriophage DNA Polymerase, *Thermus aquaticus *DNA Polymerase, proofreading Taq-Pfu blend DNA Polymerase (OptiTaq) and *Thermus flavus *DNA Polymerase exhibit a markedly lower reaction yield (Figure 3, lanes 10, 12, 14, 16). *Pyrococcus furiosus *proofreading DNA Polymerase does not result in any incorporation of BrdU; indeed, substantial DNA losses are observed (Figure 3, lane 18). The reaction is assayed for the presence of the final product, namely, the desired 977 bp band, and factors other than the ability to utilize BrdUTP are likely to play a role in the final yield. This may involve maintaining the integrity of 3-nt 5'-protruding DNA termini, owing to a lack of proofreading activities in the case of of *Taq, Tfl *and T4 polymerases. In view of the emergence of new thermostable polymerases, either discovered in nature or protein-engineered, further improvements of the method presented here are possible. Among the commercially available DNA polymerases a mutant of *Pfu *DNA polymerase that overcomes uracil stalling, allowing the polymerase to read through uracil located in the template strand or incorporated into the extending strand could be worth trying [39]. To further evaluate the universality of the method beyond just a single combination of fragments yielding a hybrid molecule, which increases the concentration of the label/per DNA molecule unit length for the purpose of HPLC analysis, additional 441 bp and 466 bp hybrid molecules were designed and constructed. The 441 bp fragment was synthesized in the single BrdU residue integration mode and also in the double BrdU incorporation mode using Bst exo^- ^DNA Polymerase (Figure 4). There was no difference in yield, regardless of whether double BrdU or single BrdU/single T residues were incorporated with a one bp precision (Figure 4, lane 3, 5). These results were confirmed with the construction of the 466 bp hybrid molecule (Figure 5, lane 9, 10). The reaction was closely monitored by the inclusion of a set of controls at key steps of the incorporation/assembly reaction (Figures 3, 4, 5). As a result of the use of non-phosphorylated primers and asymmetric cohesive ends, the synthesis reactions depicted in Figures 3, 4 and 5 gave yields of the labeled molecules of over 50% with no by-products.

In order to simplify the analysis of labeled species, we have used 441, 466 and 977 bp model hybrid molecules. However, one should keep in mind, that the upper limit of labeled molecules length is much higher. With this method, extremely long DNA fragment can be obtained by means of a multiple assembly reaction (n × maximum PCR product length: n × app. 50 kb).

The method presented therefore seems to be uniquely suited to the construction of models for *in vitro *studies of anti-cancer therapy properties. Moreover, the method can be extended to protein-DNA interaction studies, by for example, the precise incorporation of modified bases into DNA-interacting protein recognition sites present on long DNA molecules.

### Determination of the presence of BrdU in hybrid DNA molecule

**Figure 6:**
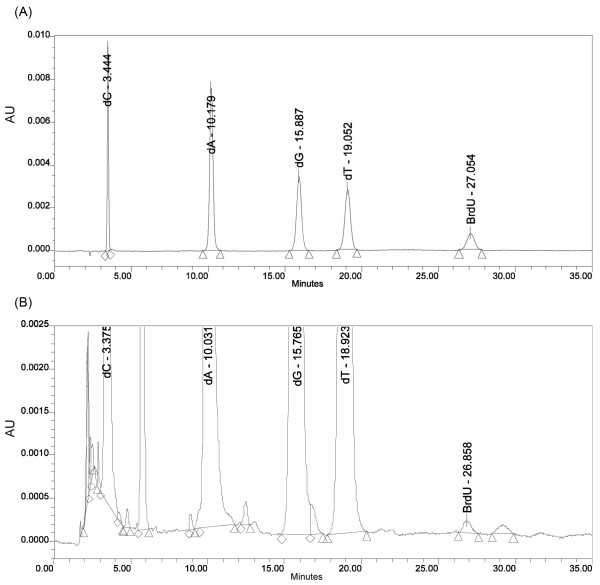
**Chromatograms of the mixture of standards (A) and BrdU-labeled 441 bp oligomer after enzymatic digestion (B)**.

The occurrence of bands corresponding to the BrdU-labeled hybrid DNA molecules in the gels (Figures 3, 4, and 5) is indirect proof that BrdU is incorporated into the ultimate product. Indeed, if the cohesive ends generated by BsaI are not filled with BrdUTP (or dTTP), no final product is obtained. However, when the partial filling of the cohesive ends precedes ligation, the formation of a hybrid molecule is highly efficient (Figure 3, lane 8; Figure 4, lanes 3, 5; Figure 5, lanes 9, 10). Thus, one can infer that if the cohesive ends are partially filled with BrdUTP, BrdU has to be present in a hybrid DNA molecule. Although this indirect reasoning sounds convincing, one can also demonstrate directly that the BrdU molecule is present in the ligation product: namely, HPLC coupled to DNA digestion down to nucleosides is a suitable method for directly demonstrating the presence of BrdU in the synthesized material. Since the sensitivity of HPLC detection depends on the amount of a substance in the assayed sample, we chose the shortest fragment (of the three synthesized) labeled with two BrdU molecules to demonstrate the presence of 5-bromouridine. As a result of this choice we analyzed a digestion mixture in which the ratio of BrdU to the remaining nucleosides was the highest possible (taking into account the fact that three DNA fragments differing in the length and number of BrdU molecules were synthesized). Figure 6 shows two chromatograms: the upper panel (Figure 6A) corresponds to the HPLC separation of a mixture containing native nucleosides and 5-bromouridine; the lower panel (Figure 6B) shows the chromatogram obtained for the digested 441 bp fragment containing two molecules of BrdU. The peak with the retention time of 26.9 min (Figure 6B) corresponds well to the BrdU signal (27.1 min) in the chromatogram of standards (Figure 6A). The small peak (see the absorbance scale) at 29.2 min, as well as the peaks before the dC peak, between the dC and dA, dA and dG and dG and dT peaks, are due to the digestion procedure, as indicated by the chromatogram obtained for the 441 bp fragment, which does not contain the BrdU molecules (not shown). Additionally, by employing the peak areas of standards and the areas of the actual signals registered for the digested 441 bp labeled oligonucleotide, we were able to calculate its base content: the amount of BrdU turned out to be equal to 1.93, which is quite close to the theoretical value of 2. HPLC separation coupled to enzymatic digestion therefore demonstrates unequivocally that an oligonucleotide containing BrdU is indeed produced within our protocol.

## Conclusions

*i*. A new method of incorporating labeled nucleotides at a precisely-predetermined location has been developed.

*ii*. The length of these enzymatically synthesized DNA molecules theoretically approaches a multiple of the maximum length obtainable in a single PCR reaction.

*iii*. The synthesis efficiency of the labeled molecules is better than 50% with no by-products

*iv*. This method is general and offers the possibility of preparing DNA duplexes labeled with a controlled number of Hal-Nbs as well as other labels.

*v*. A number of DNA polymerases have been tested for the incorporation of the haloderivative BrdU into DNA, indicating marked differences in the ability to distinguish modified dUTP from the non-modified precursor. The most efficient incorporation was obtained using Bst exo- DNA Polymerase.

*vi*. The presence of incorporated BrdU was proven by both ligation assay and HPLC analysis.

*vii*. The BrdU incorporation study is a contribution to the long-term goal of devising an anti-cancer therapy based on the *in vivo *incorporation of a haloderivative, followed by treatment with ionizing radiation.

## Methods

### Bacterial strains, media and reagents

*Escherichia coli *(*E. coli*) DH11S {*mcrA *Δ[*mrr*-*hsdRMS*(r_K-_, m_K+_)-*mcrBC*] Δ(*lac-proAB*) Δ(*recA1398*) *deoR, rpsL, srl-thi, supE*/F' *proAB*^+ ^*lacI^Q^Z*Δ*M15*} (Life Technologies, Gaithersburg, MD, USA) was used as cloning host and for DNA propagation. Bacteria were grown in 2xYT medium [40] supplemented with chloramphenicol (40 μg/ml). BrdUTP and dNTPs were from Sigma-Aldrich (St. Louis, MO, USA). Difco media components were obtained from Becton-Dickinson (Franklin Lakes, NJ, USA), agarose GTG from FMC (Rockland, MA, USA), and the DNA purification kits, T4 DNA polymerase, Tfl DNA Polymerase, Taq DNA Polymerase, OptiTaq, GeneMatrix PCR/DNA Clean-Up Purification Kit and GeneMatrix Agarose-Out DNA Purification Kit were from EURx Ltd. (Gdansk, Poland). Bst DNA Polymerase large fragment exo-, pUC19 DNA, BsaI, NcoI and BspHI, T4 DNA ligase REase were from New England Biolabs (Ipswich, MA, USA). Pfu DNA Polymerase was from Agilent Technologies, Inc. (Santa Clara, CA, USA). Oligo and custom DNA sequencing were provided by Genomed (Warsaw, Poland). All other reagents were from Amresco (Solon, OH, USA) or Sigma-Aldrich, of the highest available purity.

### Construction of DNA molecules with precisely incorporated 5-BrdU

To validate the universality of the proposed method, we used three different DNA constructs *in vitro *for the pinpoint incorporation of either double or single 5-BrdU.

#### 977 bp hybrid DNA molecule

The 977 bp hybrid DNA molecule was generated from two PCR products (379 bp and 625 bp) amplified from the pUC19 plasmid template. The 378 bp fragment was obtained using primer 1F: 5'-GTGGCGAAACCCGACAGGACT-3' and primer 1R:

5'-ATGAGTCATCCACCCAT**GAGACC**ACTTCAAGAACTC-3', whereas the primer 2F: 5'-GGATGTGCTGCAAGGCGATTA-3' and primer 2R:

5'-ATGAGTCATCCAGGGAT**GAGACC**TCTGACTTGAGCG-3' were used for the 625 bp fragment.

Incorporated BsaI sites are shown in bold and underlined. The hybrid molecule and PCR fragment sequences are available as additional files (Additional file 1).

#### 441 bp hybrid DNA molecule

The 441 bp hybrid DNA molecule was generated from two PCR products (208 bp and 260 bp) amplified from the pUC19 plasmid template. The 208 bp fragment was obtained using primer 3F: 5'-AGCGGTATCAGCTCACTCAAAGG-3' and primer 2R, whereas primer 4F: 5'- GAAGCGTGGCGCTTTCTCATAG -3' and primer 1R were used for the 260 bp fragment. The hybrid molecule and PCR fragment sequences are available as additional files (Additional file 2).

The PCR reactions for generating 977 bp and 441 bp molecules were conducted in 50 μl volume, 0.5 mM of each primer, template 2.5 ng, 60 μM dNTPs, 2.5 units Taq Polymerase under thermocycling conditions: initial denaturation at 97°C for 2.5 min, hot start at 80°C, denaturation at 94°C for 30 sec, annealing at 53°C for 30 sec, extension at 72°C for 50 sec, final flush-in at 72°C for 50 sec, 40 cycles. The amplification products were purified using the PCR/DNA Clean-Up Purification Kit.

#### 466 bp hybrid DNA molecule

The 466 bp hybrid DNA molecule was generated from two PCR products (437 bp and 480 bp) amplified from two plasmid pRZ4737-derivatives. The templates used here (pGCN1 and pGNC2) were constructed by PCR and cloning for the purpose of this work as well as further studies on protein-directed Hal-NBs incorporation. The starting DNA for the construction was modified pRZ4737 plasmid vector (Cm^R^, P15A *ori, f*1 *ori*, P_R _promoter), originally obtained from Bill Resnikoff. Two PCR fragments of 162 bp and 128 bp were amplified from the vector using oligonucleotides containing long 5'- overhangs and incorporating class-IIS REase BsaI and NcoI sites at each fragment 5'-end (forward primers) and BspHI (reverse primer). Incorporated REase sites are shown in bold or underlined. The 162 bp fragment was amplified with 5F primer: 5'- GCG**CCATGG**TCTCATCCCTGGATGACTCATTTCTTTTTTGTGC-3' and 3R primer: 5'- AGC**TCATGA**ACACCTCCTTAAAAAAAAAATGAGTCATCCATTATCACCG-3'. The 128 bp fragment was amplified with 6F primer: 5'-CTT**CCATGG**TCTCATGGGAAATCTATCACCGCA-3'and the 3R primer.

The 162 bp and 128 bp PCR products were cleaved with NcoI and BspHI and cloned into pRZ4737-derivative cleaved with BspHI. The BspHI and NcoI sites have compatible cohesive ends, which are not recleavable after cross-ligation. For further experiments plasmids were selected and designated pGCN1 and pGCN2. From these, construct upstream primers were used to amplify the 437 bp and 480 bp fragments for the labeling reaction. Primer 7F: 5'- AATTACCTATTGACGCAAGTCTCGAAGGCGACGTGCGTCC-3' was used as forward primer for the 437 bp product (template: pGCN1), and primer 8F: 5'-CATTAAATAAAGCACCAACGCCT-3' for the 480 bp product (template: pGCN2). Primer 4R: 5'-GAATTGTGAGCGGATAACAATTTCACACAG-3' was used as reverse primer for both fragments. Hybrid molecule and PCR fragment sequences are available as additional files (Additional file 3). The PCR reactions for the 437 bp and 480 bp fragments were conducted in 50 μl volume, 0.5 mM each primer, template 2.5 ng, 60 μM dNTPs, 2.5 units Taq Polymerase under thermocycling conditions: initial denaturation at 94.5°C for 1.5 min, hot start at 80°C, denaturation at 94°C for 20 sec, annealing at 58°C for 30 sec, extension at 72°C for 40 sec, final flush-in at 72°C for 50 sec, 40 cycles. Amplification products were purified using the PCR/DNA Clean-Up Purification Kit.

### BrdU labeling reactions

Purified PCR products were cleaved with BsaI (10 units per 1 μg/DNA) for 1 h at 50°C as recommended by the manufacturer. Fill-in reactions were performed in 80 μl reaction volume, containing 5 μg DNA, 40 units of polymerase, 0.2 mM BrdUTP, for 20 min at 70°C for thermophilic polymerases and 37°C for mesophilic polymerases in dedicated reaction buffers as recommended by the manufacturer. Ligation was carried out with 200 units of T4 DNA Ligase for 1.5 h at 16°C in 100 μl reaction volume in the buffer recommended by the manufacturer.

### HPLC detection of incorporated 5-BrdU

The purified DNA was digested to nucleosides prior to HPLC analysis by the simultaneous action of DNase I, snake venom phosphodiesterase (SVP) and bacterial alkaline phosphatase (BAP). 40 units of DNase I, 1 unit of BAP and 0.01 units of SVP were incubated with the purified DNA fragment in a final volume of 100 μl in the following reaction buffer: Tris-HCl (pH 8.5; 10 mM), MgCl_2 _(10 mM), CaCl_2 _(1 mM) and DTPA (diethylenetriamine pentaacetic acid, Sigma Aldrich) (40 μM). The enzymatic digestion was performed at 37°C for 3 h (1 h with each enzyme, sequentially). After digestion, reaction mixtures were purified by centrifugation using Microcon centrifugal filter units with a YM-3 membrane, NMWCO 3 kDa (Millipore). The resulting samples were dried down by rotary evaporation in a Speed-Vac Concentrator and dissolved in 60 μl of ultrapure water. 20 μl of the filtered sample was subjected to HPLC analysis, which was performed on a Waters^® ^600E Delivery System with a Waters^® ^2487 Dual Lambda Absorbance detector set at 260 nm for monitoring the effluents. A Waters^® ^Atlantis reverse-phase dC_18 _column (4.6 × 150 mm; 5 μm in particle size and 100 L in pore size) with a mobile phase consisting of deionized water, acetonitrile (Sigma-Aldrich, Poland) and 1% formic acid (POCH S.A., Poland) (pH 2.55; 87.7:2:10.3, v/v/v) was used. The flow rate was set at 1 ml/min.

## Authors' contributions

IS performed most of the experiments, participated in the preparation of the figures and in the design and interpretation of all the experimental analyses. KP performed some experiments and participated in the design and interpretation of the experimental analyses. AŻS participated in the design and interpretation of the experimental analyses, the preparation of the figures and the writing of the manuscript. JJF prepared the cloned templates. PMS came up with the concept of the new method of nucleobase incorporation at a precisely pre-determined distance, participated in the design and interpretation of experiments and drafted the manuscript. JR participated in the design and interpretation of the experiments, conceived the project, coordinated its execution and drafted the manuscript. All authors read and approved the final manuscript.

## Supplementary Material

Additional file 1 **Sequence of the 977 hybrid DNA molecule**. All DNA sequences are written in the 5'-3' direction. The DNA sequence of the 379 bp PCR fragment is written in capital letters. The DNA sequence of the 625 bp PCR fragment is written in small letters. The positions that can be substituted with BrdU are marked in red and bold. The arrows show the BsaI cleavage points. The BsaI recognition sequences are placed in rectangular boxes. The BsaI restriction fragments forming the hybrid molecule are underlined. The DNA fragments removed following BsaI cleavage are in italics. Click here for file

Additional file 2 **Sequence of the 441 hybrid DNA molecule**. All DNA sequences are written in the 5'-3' direction. The DNA sequence of the 260 bp PCR fragment is written in capital letters. The DNA sequence of the 208 bp PCR fragment is written in small letters. The positions that can be substituted with BrdU are marked in red and bold. The arrows show the BsaI cleavage points. The BsaI recognition sequences are placed in rectangular boxes. The BsaI restriction fragments forming the hybrid molecule are underlined. The DNA fragments removed following BsaI cleavage are in italics. Click here for file

Additional file 3 **Sequence of the 446 hybrid DNA molecule**. All DNA sequences are written in the 5'-3' direction. The DNA sequence of the 437 bp PCR fragment is written in capital letters. The DNA sequence of the 480 bp PCR fragment is written in small letters. The positions that can be substituted with BrdU are marked in red and bold. The arrows show the BsaI cleavage points. The BsaI recognition sequences are placed in rectangular boxes. The BsaI restriction fragments forming the hybrid molecule are underlined. The DNA fragments removed following BsaI cleavage are in italics. Click here for file
