# Supplementary figures and images for: Enzymatic synthesis of long double-stranded DNA labeled with haloderivatives of nucleobases in a precisely pre-determined sequence
Source: BMC Biochem. 2011 Aug 24;12:47. doi: 10.1186/1471-2091-12-47 (PMC3179937; doi:10.1186/1471-2091-12-47)

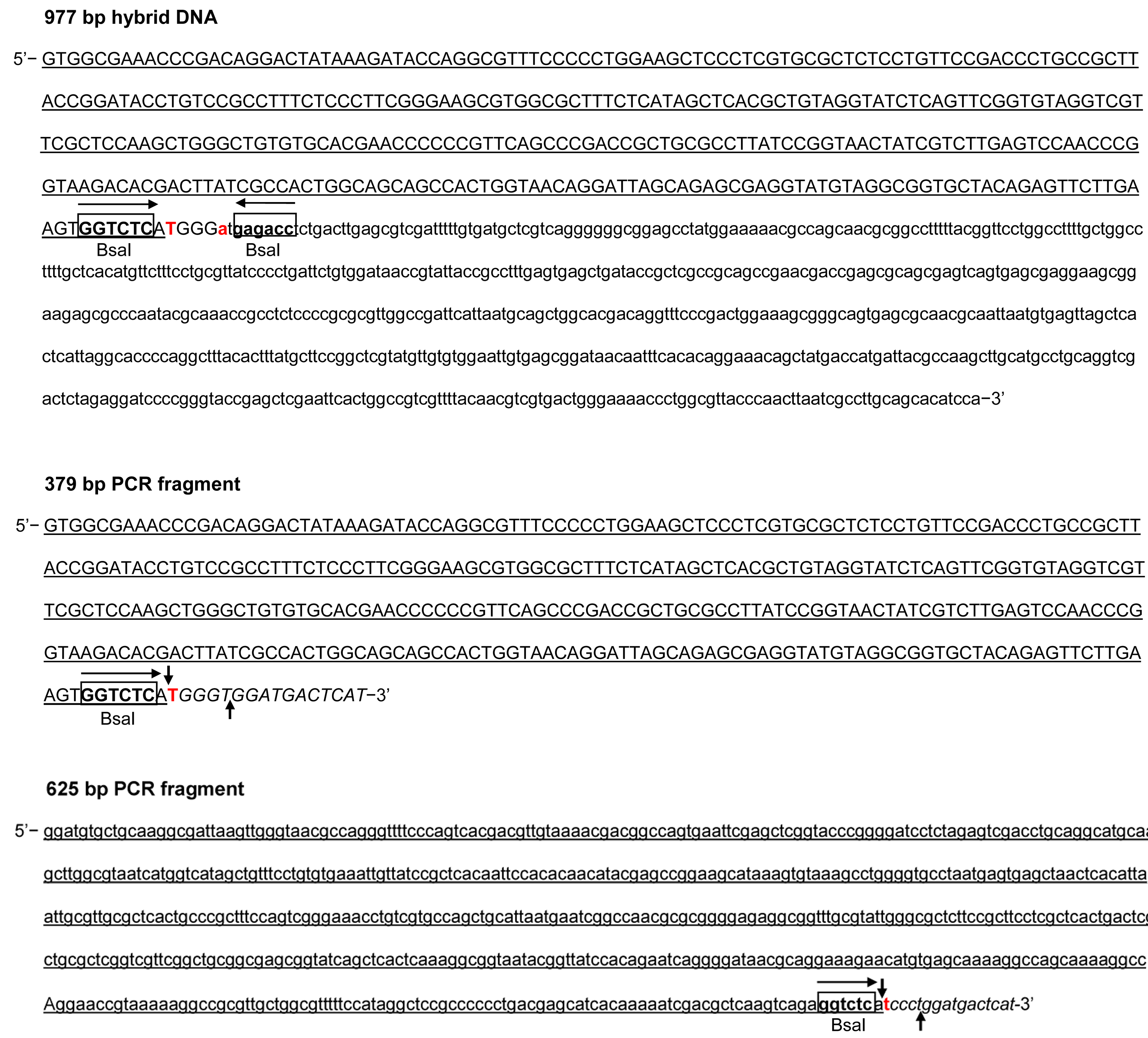

Supplement: Additional file 1 — Sequence of the 977 hybrid DNA molecule. All DNA sequences are written in the 5'-3' direction. The DNA sequence of the 379 bp PCR fragment is written in capital letters. The DNA sequence of the 625 bp PCR fragment is written in small letters. The positions that can be substituted with BrdU are marked in red and bold. The arrows show the BsaI cleavage points. The BsaI recognition sequences are placed in rectangular boxes. The BsaI restriction fragments forming the hybrid molecule are underlined. The DNA fragments removed following BsaI cleavage are in italics. [file 1471-2091-12-47-S1.TIFF]

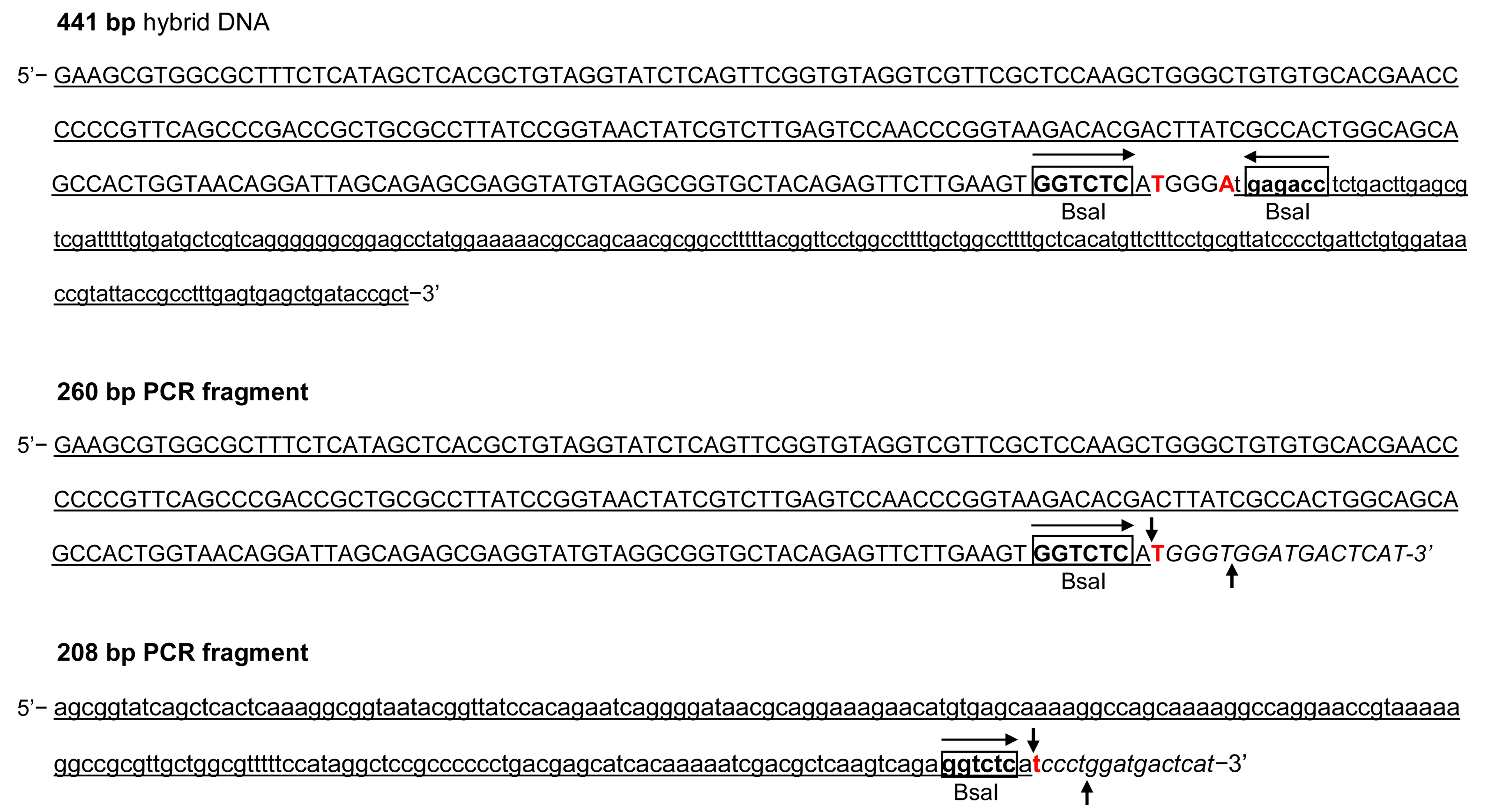

Supplement: Additional file 2 — Sequence of the 441 hybrid DNA molecule. All DNA sequences are written in the 5'-3' direction. The DNA sequence of the 260 bp PCR fragment is written in capital letters. The DNA sequence of the 208 bp PCR fragment is written in small letters. The positions that can be substituted with BrdU are marked in red and bold. The arrows show the BsaI cleavage points. The BsaI recognition sequences are placed in rectangular boxes. The BsaI restriction fragments forming the hybrid molecule are underlined. The DNA fragments removed following BsaI cleavage are in italics. [file 1471-2091-12-47-S2.TIFF]

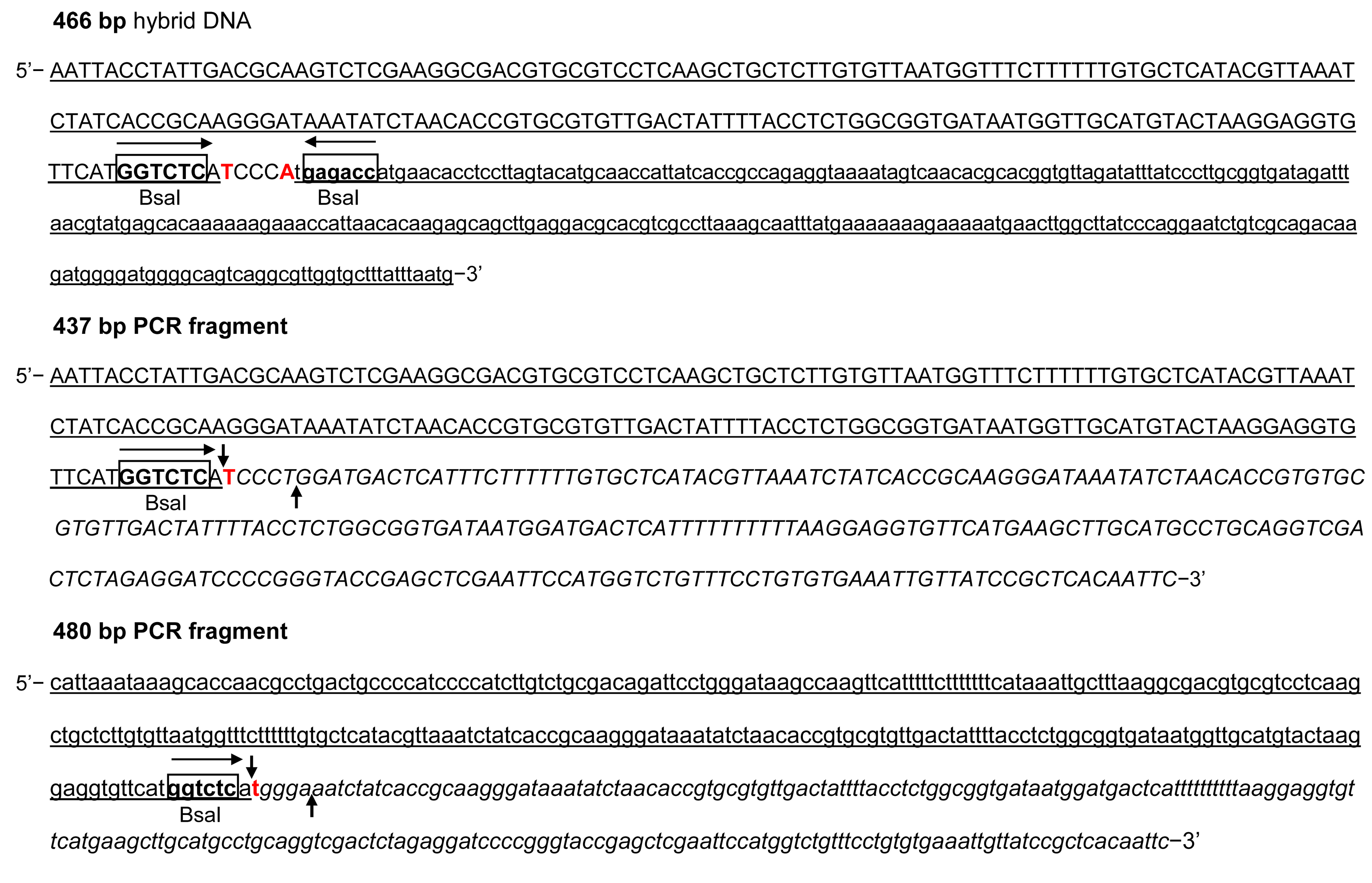

Supplement: Additional file 3 — Sequence of the 446 hybrid DNA molecule. All DNA sequences are written in the 5'-3' direction. The DNA sequence of the 437 bp PCR fragment is written in capital letters. The DNA sequence of the 480 bp PCR fragment is written in small letters. The positions that can be substituted with BrdU are marked in red and bold. The arrows show the BsaI cleavage points. The BsaI recognition sequences are placed in rectangular boxes. The BsaI restriction fragments forming the hybrid molecule are underlined. The DNA fragments removed following BsaI cleavage are in italics. [file 1471-2091-12-47-S3.TIFF]
